# On the efficiency of paired ranked set sampling for estimating the population mean in the presence of non-response

**DOI:** 10.1038/s41598-024-76072-y

**Published:** 2024-10-25

**Authors:** Syed Abdul Rehman, Laila A. Al-Essa, Javid Shabbir, Zaheen Khan

**Affiliations:** 1 Department of Statistics, Quaid-i-Azam University, Islamabad, 45320 Pakistan; 2 Department of Mathematical Sciences, College of Science, Princess Nourah bint Abdulrahman University, Riyadh, 11671 Saudi Arabia; 3 Department of Statistics, University of Wah, Wah Cantt, 47040 Pakistan; 4 Department of Statistics, Federal Urdu University of Arts, Science and Technology, Islamabad, 44000 Pakistan

**Keywords:** Scientific data, Statistics

## Abstract

Non-response is a common problem faced by surveyors while conducting surveys; this introduces a potential bias in the estimates of population parameters. One method of dealing with non-response is subsampling of the non-respondents, which increases precision in estimates by increasing the sample size. This study proposes an unbiased mean estimator in the presence of non-response using the Paired Ranked Set Sampling (PRSS) technique. The proposed estimator is based on a suggested strategy for adapting the subsampled units into the initial sample. Variance of the proposed estimator is derived, and conditions are provided for which the proposed estimator performs better than existing estimators. We conduct a simulation study to evaluate the precision of the proposed estimator in comparison with other existing estimators for estimating the population mean. In simulation, we assume populations based on normal distribution, exponential distribution, and real-life data on abalone. Simulation results show that the proposed mean estimator exhibits a higher probability of precisely estimating the finite population mean.

## Introduction

Estimation of population parameters based on sample data is an important statistical technique. Though Simple Random Sampling (SRS) is a simple and straightforward method to collect data, estimates based on SRS are thought to be imprecise when the population under study is not symmetric. This suggests that the sample selection procedure has a greater impact on statistical estimates and inferences. Experts recommend sampling techniques other than SRS in such situations. Rank Set Sampling (RSS) is one efficient technique among these alternative sampling methods; it examines a large number of population units based on their relative sizes to draw a smaller sample. The RSS increases the precision of estimates by reducing sampling errors. The RSS sampling procedure also has the advantage that it can utilize the ranks of a closely related concomitant variable to collect data at the data collection stage. This method was introduced by^1^, and it is more suitable in the environmental sciences, where observing a sample unit is costly or challenging, but ranking units based on visual assessments is easy.^2^ developed an unbiased estimator of the population mean using the RSS technique.^3^ introduced the use of a closely related concomitant variable for ranking the units of the study variable. Later on, the Median RSS (MRSS) and Paired RSS (PRSS) methods were suggested by^4^ and^5^ respectively. The methods of Double RSS (DRSS), Double MRSS (DMRSS), and Double PRSS (DPRSS) were suggested by^6,7^ and^8^ respectively. Further expansions and reviews on this topic can be found in^9–12^, and^13^ etc.

On the other hand, non-response is one major issue faced by the surveyors while surveying the population. It refers to a situations when one or more units have fully or partially failed to respond to the survey. In other words, it is the inability to obtain a sufficient response from all survey items in a given sampling frame. Non-response introduces bias in the estimates of population parameters when it occurs in a systematic pattern. A lower response rate can increase the risk and magnitude of non-response bias, while it has no greater impact on the estimates when the data is missing at random (MAR). In the social sciences, non-response can be associated with certain ethnic, demographic, political, or religious groups. The sample becomes unrepresentative, and it may not fairly represent the opinion and traits of that group. This can compromise the validity of the survey findings, making it challenging to generalize the results to a broader population. For example, in the biological sciences, marine organisms such as fish and invertebrates may run away or behave evasively when approached by researchers, making it difficult to obtain measurements about their size, height, weight, etc. Non-response has the disadvantage of reducing the effective sample size, which leads to inaccurate statistical estimates. The literature offers a number of strategies to reduce the impact of non-response. Although list-wise and pair-wise deletion is frequently used, it is not considered an effective solution. The alternative solutions are subsampling of non-responding units and imputation of the mean. Subsampling of non-responding units increases the response rate and thus improves the accuracy of estimates. The idea of subsampling was suggested by^14^, it is called the naive approach. Though references are available for additional research that improved on the naive approach, a detailed study was presented recently by^15^ and^16^. They employed various RSS methods to handle non-responses through subsampling and imputations.

Motivated by the importance of this topic, in this paper we suggest a new method for data collection, assuming non-response is anticipated while conducting surveys. We explain the procedure of adjusting sabsampled units into the initial sample and develop a novel mean estimator. Our objective is to contribute to the literature on RSS theory by suggesting an efficient estimator of the finite population mean in the presence of on-response. This study begins with an overview of the preliminary concepts in “Order statistics in surveys” section. “Existing methods and estimators” section reviews the current subsampling techniques and their corresponding mean estimators. In “Proposed methodology and mean estimator” section, we describe the proposed method for obtaining an initial sample in the presence of non-response and the procedure for adapting subsampled units into the initial sample is also explained. Additionally, the proposed mean estimator and its variance are also provided in “Proposed methodology and mean estimator” section, and the conditions under which the suggested estimator outperforms the existing estimators are provided. The procedure for simulation study and its results are presented in “Simulation study” section, while the concluding remarks are presented in “Application to real data” section.

## Order statistics in surveys

In this section, we discuss the theory of order statistics, which involves sorting the data points within a sample. Order statistics assist in enhancing the precision of estimates by utilizing information obtained from ranking the data points. Additionally, we explain the procedures of RSS and PRSS for gathering data, which rely on the principles of order statistics.

### Ordered statistics

Consider a random variable *Z* for which we have a sample ${Z_k}; k = 1,\ldots,n$ of size *n* distributed identically and independently, then the $k^{th}$ smallest value is defined as the $k^{th}$ order statistic. Generally, ordered statistics of *Z* are written as ${Z_{\left( 1 \right) }} \leqslant {Z_{\left( 1 \right) }} \leqslant \cdots \leqslant {Z_{\left( n \right) }}$, but some of the ordered values can coincide. The likelihood of such a coincidence approaches zero if $Z_k$ is continuous and has a non-negative density. Assuming the sample comes from a continuous distribution such that all observed values are distinct, i.e., the inequalities ${Z_{\left( 1 \right) }}< {Z_{\left( 1 \right) }}<\cdots < {Z_{\left( n \right) }}$ hold, the pdf and CDF of $k^{th}$ order statistics are given by

1 $$\begin{aligned} {f_{Z\left( k \right) }}\left( z \right) = \frac{{n!}}{{\left( {n - k} \right) !\left( {k - 1} \right) !}}{\left\{ {{F_Z}\left( z \right) } \right\} ^{k - 1}}{\left\{ {1 - {F_Z}\left( z \right) } \right\} ^{n - k}}{F_Z}\left( z \right) , \end{aligned}$$

and

2 $$\begin{aligned} {F_{Z\left( k \right) }}\left( z \right) = \sum \limits _{i = k}^n {{{\left\{ {{F_Z}\left( z \right) } \right\} }^j}{{\left\{ {1 - {F_Z}\left( z \right) } \right\} }^{n - j}}}, \end{aligned}$$

where ${{F_Z}\left( z \right) }$ is the CDF of the underlying distribution. Although, the mean and variance based on ordered statistics are the same as for unsorted observations, but when the information gained from the ranking of sample units is used wisely, the population parameters can be estimated more precisely.

### Ranked set sampling (RSS)

This method utilizes the information provided by the ranking of the sample units and thus estimates the population mean more precisely than the SRS method. The procedure for sampling through RSS involves examining $m^2$ units from the population and distributing them into *m* sets, each of size *m*. Rank the units within each set using some visual judgments or any other inexpensive method. We select a sample of *m* units such that the $i^{th}$ smallest unit from the $i^{th}$ set is chosen. We repeat this procedure *r* times to obtain a final sample of size $n = rm$. The selected sample can be represented as $S = \left\{ {{Z_{i\left( i \right) j}}} \right\} _{i = 1}^m$ such that $E\left( {{Z_{i\left( i \right) j}}} \right) = {\mu _{\left( i \right) z}}$ and $Var\left( {{Z_{i\left( i \right) j}}} \right) = \sigma _{\left( i \right) z}^2$, where ${{Z_{i\left( i \right) j}}}$ shows $i^{th}$ order statistics in the $i^{th}$ set of $j^{th}$ cycle. The sample mean based on this method is given by

3 $$\begin{aligned} \hat{\bar{Z}}_{rss} = \frac{1}{{rm}}\sum \limits _{j = 1}^r {\sum \limits _{i = 1}^m {{Z_{i\left( i \right) j}}} } \end{aligned}$$

The estimator $\hat{\bar{Z}}_{rss}$ is unbiased whereas its variance is given by

4 $$\begin{aligned} Var\left( \hat{\bar{Z}}_{rss} \right) = \frac{{\sigma _z^2}}{n} - \frac{1}{{nm}}\sum \limits _{i = 1}^m {\Delta _{\left( i \right) }^2} \end{aligned}$$

where ${\Delta _{\left( i \right) }} = {\mu _{\left( i \right) z}} - {\mu _z}$ shows deviation of $i^{th}$ order statistics mean from overall population mean ${\mu _z}$. In Equation (3), the term ${{\sigma _z^2} / n}$ is variance of sample mean based on SRS sample of size *n*. The term ${\left( {nm} \right) ^{ - 1}}\sum \nolimits _{i = 1}^m {\Delta _{\left( i \right) }^2}$ A is a positive quantity that shows the magnitude of the gain in efficiency for estimating the population mean using RSS instead of SRS sampling. This increase in efficiency is due to the information provided by ordering the sample units.

### Pair ranked set sampling (PRSS)

This method of RSS sampling was proposed by^4^ while considering the cost of examining units for ranking. This method examines a smaller number of observations as compared to the RSS method. Data collection through this approach is different for even and odd sample sizes *m*, described as:

- If *m* is even, examine ${{{m^2}} / 2}$ units from the population and allocate them into *m*/2 independent sets each of size *m*. Rank the units within each set and select the pair of $i^{th}$ and $(m+1-i)^{th}$ units from all sets, which provides a sample denoted as $S = \left\{ {\left( {{Z_{i\left( i \right) j}},{Z_{i\left( {m + 1 - i} \right) j}}} \right) _{i = 1}^{\frac{m}{2}}} \right\}$.
- If *m* is odd, examine $\left( {{{m\left( {m + 1} \right) } / 2}} \right)$ units from the population and distribute them into ${{\left( {m + 1} \right) } / 2}$ independent sets each of size *m*. Rank the units within each set and select the pair of $i^{th}$ and $(m+1-i)^{th}$ units from the first ${{\left( {m - 1} \right) } / 2}$ sets, while ${\left( {{{\left( {m + 1} \right) } / 2}} \right) ^{th}}$ unit is selected from the last set. The selected sample can be represented as $S = \left\{ {\left( {{Z_{i\left( i \right) j}},{Z_{i\left( {m + 1 - i} \right) j}}} \right) _{i = 1}^{\frac{{m - 1}}{2}},{Z_{m\left( {\frac{{m + 1}}{2}} \right) j}}} \right\}$.

A final sample of size $n=rm$ can be obtained by repeating this procedure *r* times. The estimator of the population mean for this method is given by

5 $$\begin{aligned} \hat{\bar{Z}}_{prss}=\frac{1}{{rm}} \sum \limits _{j = 1}^r {\left\{ \begin{array}{ll} {\sum \limits _{i = 1}^{\frac{m}{2}} {\left( {{Z_{i\left( i \right) j}} + {Z_{i\left( {m + 1 - i} \right) j}}} \right) } } & \text {if }\, m\, \text {is even} \\ \sum \limits _{i = 1}^{\frac{{m + 1}}{2}} {{Z_{i\left( i \right) j}} + \sum \limits _{i = 1}^{\frac{{m - 1}}{2}} {{Z_{i\left( {m + 1 - i} \right) j}}} } & \text {if}\, m\, \text {is odd} \\ \end{array}\right. } \end{aligned}$$

The estimator $\hat{\bar{Z}}_{prss}$ is unbiased whereas its variance is given by

6 $$\begin{aligned} V(\hat{\bar{Z}}_{prss})= \frac{{\sigma _z^2}}{{n}} - \frac{1}{{nm}} {\left\{ \begin{array}{ll} \sum \limits _{i = 1}^{\frac{m}{2}} {\left( {{\Delta _{\left( i \right) }} + {\Delta _{\left( {m + 1 - i} \right) }}} \right) } + 2\sum \limits _{i = 1}^{\frac{m}{2}} {{\sigma _{z\left( {i,m + 1 - i} \right) }}} & \text {if}\, m\, \text {is even} \\ \sum \limits _{i = 1}^{\frac{{m + 1}}{2}} {{\Delta _{\left( i \right) }} + \sum \limits _{i = 1}^{\frac{{m - 1}}{2}} {{\Delta _{\left( {m + 1 - i} \right) }}} + 2\sum \limits _{i = 1}^{\frac{{m - 1}}{2}} {{\sigma _{z\left( {i,m + 1 - i} \right) }}} } & \text {if}\, m\, \text {is odd}, \\ \end{array}\right. } \end{aligned}$$

where ${{\sigma _{z\left( {i,m+1-i} \right) }}}$ is covariance between $i^{th}$ and $(m+1-i)^{th}$ ordered statistics. The Equation (6) shows that sample mean based on PRSS is at least as efficient as sample mean based on SRS. The efficiency of PRSS depends on terms $\Delta _{(i)}$, $\Delta _{(m+1-i)}$ and ${{\sigma _{z\left( {i,m+1-i} \right) }}}$, which is due to ranking of the units.

### The perfect and imperfect ranking

If ranking is based on the characteristics of the study itself, it is called a perfect ranking. This can be done by means of visual judgments of the surveyor. Though this type of ranking is easy and inexpensive, and one can reasonably assume that ranking is free from error, there is still a possibility that an error in ranking might occur due to a mistake in human judgments. In situations where perfect ranking is not feasible or possible,^3^ proposed an alternative method of ranking the units. He ranked units of the study variable using the order of a closely related auxiliary variable, assuming that information is available or can easily be obtained on the auxiliary variable. He referred to this imperfect ranking and the theory of RSS. Some researchers referred to this ranking with errors since it is possible to place a greater unit before any smaller unit when units are observed. This type of error might cause a decline in the efficiency of estimates based on order statistics. In our study, we considered both types of ranking when developing survey methods and deriving their corresponding mean estimators.

## Existing methods and estimators

In this section, we review the naive method of subsampling in the presence of non-response. We also discuss two more recently proposed approaches that utilize order statistics and RSS to increase the efficiency of estimates in the presence of non-responses. The subsequent mean estimators for each approach and their variance are also provided.

### The naive approach

The problem of subsampling under non-responses was suggested by^14^. They justified that subsampling can take into account a part from non-respondents; this can reduce non-response bias and increase the overall sample size. The procedure for this method is described as follows:

Consider we aim to estimate the population mean, for which we collect a sample of size $n_s$ using a simple random sample without replacement (SRSWOR), represented as $S = \left\{ {{Z_i};i = 1,\ldots,{n_s}} \right\}$. Assume that $n_{1}$ units respond to the survey, i.e., ${S_1} = \left\{ {{Z_{1i}};i = 1,\ldots,{n_1}} \right\}$, while ${n_2}=n-{n_1}$ units failed to respond. This allows us to divide the population into two groups called response and non-response groups of size $N_1$ and $N_2$ respectively. We want to include some units from the non-response group; for this purpose, we collect a subsample from non-responding units such that special efforts are made to include a part (say $n'_2; k=n_2/n'_2$) of non-respondents forming ${S_2} = \left\{ {{Z_{2i}};i = 1,\ldots,{n'_2}} \right\}$. Thus, the total sample in hand is $n=n_1+n'_2$, based on which^14^ proposed the following estimator of population mean:

7 $$\begin{aligned} \hat{\bar{Z}}_{HH} = {w_1}{{\bar{z}}_{1srs}} + {w_2}{{\bar{z}}_{1srs}}, \end{aligned}$$

where

$$\begin{aligned} {{\bar{z}}_{1srs}} = \frac{1}{{{n_1}}}\sum \limits _{i = 1}^{{n_1}} {{Z_{1i}}} ,\hspace{0.2cm} {{\bar{z}}_{2srs}} = \frac{1}{{{n'_2}}}\sum \limits _{i = 1}^{{n'_2}} {{Z_{2i}}} ,\hspace{0.2cm} \mathrm{ }{w_i} = \frac{{{n_i}}}{n}\mathrm{{;\hspace{0.2cm} }}i = 1,2. \end{aligned}$$

The estimator $\hat{\bar{Z}}_{HH}$ is unbiased, however its variance is given by

8 $$\begin{aligned} V\left( \hat{\bar{Z}}_{HH}\right) = {\lambda _n}\sigma _z^2 + \frac{{{W_2}\left( {K - 1} \right) }}{n}\sigma _{z2}^2, \end{aligned}$$

where

$$\begin{aligned} \lambda = \frac{{N - n}}{{Nn}},\hspace{0.1cm} {W_i}=\frac{{{N_i}}}{N},\hspace{0.1cm} \sigma _z^2 = \frac{1}{{N - 1}}\sum \limits _{i = 1}^N {{{\left( {{Z_i} - {\mu _z}} \right) }^2}} ,\hspace{0.1cm} \sigma _{z2}^2 = \frac{1}{{{N_2} - 1}}\sum \limits _{i = 1}^{{N_2}} {{{\left( {{Z_{2i}} - {\mu _z}_2} \right) }^2}} . \end{aligned}$$

### Bouza (2013) approach

Keeping in mind the efficient nature of RSS sampling,^15^ proposed an estimator of population mean that involves the collection of subsamples from non-response groups. He proved that this estimator is at least as efficient as the naive estimator. The procedure under this approach is similar to that described for the naive approach, except for collecting subsample from non-responding units. He suggested collecting a sample of size $n'_2 \cong rm$ using the RSS method instead of the SRS method and suggested the following estimator:

9 $$\begin{aligned} \hat{\bar{Z}}_{B} = {w_1}{{\bar{z}}_{1srs}} + {w_2}{{\bar{z}}_{2rss}}, \end{aligned}$$

where ${{\bar{z}}_{1srs}}$ is sample mean based on $n_1$ units collected from response group, whereas ${{\bar{z}}_{2rss}}$ is sample mean calculated based on $n'_2$ units of non-response group collected through RSS sampling method as explained in Section (). The estimator $\hat{\bar{Z}}_{B}$ is unbiased, while its variance is given by

10 $$\begin{aligned} V\left( \hat{\bar{Z}}_{B} \right) = \frac{{\sigma _z^2}}{n} + \frac{{{W_2}\left( {K - 1} \right) \sigma _{z2}^2}}{n} - \frac{{{W_2}K}}{{nm}}\sum \limits _{i = 1}^m {\Delta _{2\left( i \right) }^2}, \end{aligned}$$

where ${\Delta _{2\left( i \right) }} = {\mu _{2\left( i \right) z}} - {\mu _{2z}}$, such that ${\mu _{2\left( i \right) z}}$ is mean of $i^{th}$ order statistics and ${\mu _{2z}}$ is overall mean of non-response group. Equation (10) shows that the estimator $\hat{\bar{Z}}_{B}$ is at least as efficient as the mean estimator $\hat{\bar{Z}}_{HH}$ of the naive method. The magnitude of efficiency gained by the estimator $\hat{\bar{Z}}_{B}$ is due to factor $\sum \nolimits _{i = 1}^m {\Delta _{2\left( i \right) }^2}$ which is due to the order statistics at second attempt. If ${\mu _{2\left( i \right) z}} \ne {\mu _{2z}}$, then it is straightforward to achieve $V\left( \hat{\bar{Z}}_{B} \right) < V\left( \hat{\bar{Z}}_{HH} \right)$.

### Fatima (2022) approach

Recently, Fatima extended on the concept of^14^ and proposed an estimator of the population mean such that data collection is done using the RSS method on both attempts. This approach is somehow different from other approaches that require some explanation. The procedure for this approach is described below as follows:

Consider the^14^ naive approach where population $\varphi$ can be divided into response group $\varphi _1$ and non-response group $\varphi _2$. Assume that information on $Z_i$ is available in the first attempt from $\varphi _1$, while information about $Z_i$ is not available in $\varphi _1$ at the first attempt; however, information on $Z_i$ is available in $\varphi _2$ at the second attempt only. Let $s_j$ denote sample at $j^{th}$ draw such that $s_j=s_1+s_2$, where ${s_1} \subset {\varphi _1}$ and ${s_2} \subset {\varphi _2}$. Using the RSS method described in Section (), a sample of size $n_s=rm$ is initially observed and information on *Z* is recorded. Assuming a complete response to $s_1$ of size $n_1$, we repeat the additional steps of RSS to draw a subsample $s'_2$ of size $n'_2 \cong r_2 m$ such that ${s'_{2(j)}} = {{{s_{2(j)}}} / {{k_{(j)}}}}$, where ${k_{(j)}} > 1$, and obtain a response for *Z*. Let ${\bar{z}_{1\left( i \right) }}$ and ${\bar{z}_{2\left( i \right) }}$ be the sample means of the study variable based on the samples $s_1$ and $s_2$, respectively, then ${\bar{z}'_{2\left( j \right) }}$ is the mean of the subsample collected from $s_2$. The weighted estimator of the population mean is

11 $$\begin{aligned} \bar{z}_{\left( i \right) } = {\omega _{1\left( i \right) }}{\bar{z}_{1\left( i \right) }} + {\omega _{2\left( i \right) }}{\bar{z}'_{2\left( i \right) }}, \end{aligned}$$

where ${\omega _i} = {{{m _i}} / m }$. Thus, Estimator of population mean based on this approach is given by

12 $$\begin{aligned} \hat{\bar{Z}}_F = \frac{1}{m}\sum \limits _{i = 1}^m {{{\bar{z}}_{\left( i \right) }}} \end{aligned}$$

Estimator $\hat{\bar{Z}}_F$ is unbiased whereas its variance is given by

13 $$\begin{aligned} V\left( \hat{\bar{Z}}_F \right) = \frac{{\sigma _z^2}}{n} + \frac{{\sigma _z^2}}{{nm}}\sum \limits _{i = 1}^m {{\omega _{2\left( i \right) }}\left( {{k_{\left( i \right) }} - 1} \right) - \frac{1}{{nm}}} \sum \limits _{i = 1}^m {{\omega _{2\left( i \right) }}} \left( {{k_{\left( i \right) }} - 1} \right) \Delta _{2\left( i \right) }^2 \end{aligned}$$

## Proposed methodology and mean estimator

Motivated by the cost-effective strategy of the PRSS technique and the informative nature of utilizing order statistics, we suggest a new sampling method based on the assumption that non-response is expected when surveying the target population. This method adapts the subsampling units into the initial sample in such a way that each ordered statistic can be used multiple times depending on the nature of the non-response occurred in the initial sample. The procedure for this method is described as follows:

Initially, repeat the PRSS procedure *r* times to select *m* units in each cycle from the population. This allows to allocate these units into a table of *m* columns and *r* rows such that the *m* columns display the $$i^{th}; i=1,..,m$$ ordered statistics selected in the $$j^{th}; j=1,\ldots,r$$ cycle. Observe values from the selected units and identify missing observations. Let $$Z_{(i)j}$$ show the response is obtained for the $$i^{th}$$ ordered statistics in the $$j^{th}$$ cycle, while $$Z_{\left( i \right) j}^*$$ indicates the same unit when it fails to respond. We collect a subsample of *m* units in the second attempt using the PRSS method and collect responses from them. Let $$Z_{\left( i \right) }^{**}$$ show the $$i^{th}$$ ranked value from the subsample drawn at the second attempt. This value is imputed into all the missing cells of the corresponding $$i^{th}$$ order statistics of the initial table. This method allows for the utilization of a single subsampled value more than once in the table of the initial sample. This results in completing the initial table as if the responses were collected from all units in the initial sample. The information provided by ordered statistics assists in allocating the subsampled values in the initial table suitably, such that appropriate weights for every subsampled value are obtained.

**Fig. 1:**
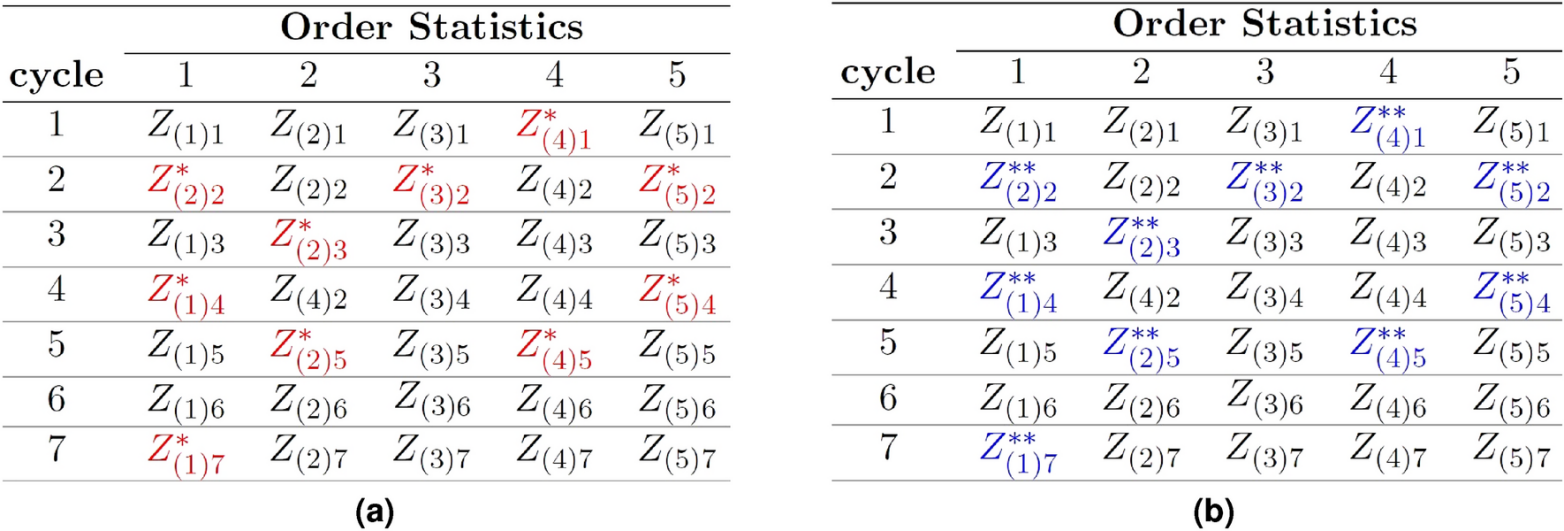
Initial table; before and after replacing the subsampling units. (**a**) Before subsampling. (**b**) After subsampling.

Figure 1 shows an exemplary illustration of the proposed survey method for set size $m=5$ and number of cycles $r=7$

Let $${Y_j} = \left\{ {{Z_{1\left( 1 \right) j}},{Z_{2\left( 2 \right) j}},\ldots,{Z_{2\left( {m - 1} \right) j}},{Z_{1\left( m \right) j}}} \right\}$$ denote the units responded at first attempt of PRSS sample in the $$j^{th}$$ cycle. Also, let $${Y^*} = \left\{ {Z_{1\left( 1 \right) }^{**},Z_{2\left( 2 \right) }^{**},\ldots,Z_{2\left( {m - 1} \right) }^{**},Z_{1\left( m \right) }^{**}} \right\}$$ shows the subsample of size *m* collected at second attempt using PRSS method, then the proposed mean estimator is given by

14 $$\begin{aligned} \hat{\bar{Z}}_{R} = \frac{1}{{rm}}\sum \limits _{i = 1}^m {\left( {\sum \limits _{j = 1}^{{r_{\left( i \right) }}} {{Y_j}} + \left( {r - {r_{\left( i \right) }}} \right) {Y^*}} \right) }, \end{aligned}$$

where ${{r_{\left( i \right) }}}$ shows the number of observations in the $i^{th}$ observation that responded at first attempt. The estimator $\hat{\bar{Z}}_{R}$ is unbiased, whereas its variance is given by.

15 $$\begin{aligned} V\left( \hat{\bar{Z}}_{R} \right) = {\left\{ \begin{array}{ll} \frac{1}{{nm}}\sum \limits _{i = 1}^m {\left( {{P_{\left( i \right) }}\sigma _{z\left( i \right) }^2 + rQ_{\left( i \right) }^2\sigma _{z\left( i \right) }^{*2}} \right) + \frac{2}{{{r^2}}}} \sum \limits _{i = 1}^{\frac{m}{2}} {\left( {{\sigma _{z\left( {i,m + 1 - i} \right) }} + {r^2}Q_{\left( i \right) }^2\sigma _{z\left( {i,m + 1 - i} \right) }^*} \right) } & \text {if}\, m\, \text {is even} \\ \frac{1}{{nm}}\sum \limits _{i = 1}^m {\left( {{P_{\left( i \right) }}\sigma _{z\left( i \right) }^2 + rQ_{\left( i \right) }^2\sigma _{z\left( i \right) }^{*2}} \right) + \frac{2}{{{r^2}}}} \sum \limits _{i = 1}^{\frac{{m - 1}}{2}} {\left( {{\sigma _{z\left( {i,m + 1 - i} \right) }} + {r^2}Q_{\left( i \right) }^2\sigma _{z\left( {i,m + 1 - i} \right) }^*} \right) } & \text {if}\, m\, \text {is odd} \\ \end{array}\right. } \end{aligned}$$

where $P_{(i)}=r_{(i)}/r$ is probability of obtaining response for the $i^{th}$ ordered unit at first attempt, whereas ${Q_{(i)}} = 1 - {P_{(i)}}$. The term ${\sigma _{z\left( i \right) }^2}$ is the variance of the $i^{th}$ order statistics, while ${{\sigma _{z\left( {i,m + 1 - i} \right) }}}$ is the covariance between the $i^{th}$ and $(m+1-i)^{th}$ order statistics responded on the first attempt. Similarly, ${\sigma _{z\left( i \right) }^{*2}}$ is variance of $i^{th}$ order statistics, and ${\sigma _{z\left( {i,m + 1 - i} \right) }^*}$ is covariance between the $i^{th}$ and $(m+1-i)^{th}$ order statistics collected from the subsample. Equation (15) shows that the proposed estimator is also consistent in nature.

If a complete response is obtained on the first attempt, then Equation (15) transforms into Equation (VPRSS), which is the variance of the mean estimator of the PRSS sample based on $n=rm$ units. Similarly, if all units failed to respond on the first attempt and only a subsample is considered for estimation, then Equation (15) transforms into the variance of the mean estimator of a PRSS subsample of size *m*. Our suggested estimator is expected to estimate the population mean more precisely in situations where the covariance between the lower and higher order statistics in the data is negative. This happens when the underlying population tends to yield larger values as the sample proceeds. The lower-order statistics may tend to be smaller, while the later ones tend to be larger. This inverse relationship can result in negative covariance among certain pairs of order statistics.

In the case of an imperfect ranking, the proposed mean estimator and its variance can be written as

16 $$\begin{aligned} \hat{\bar{Z}}_R = \frac{1}{{rm}}\sum \limits _{i = 1}^m {\left( {\sum \limits _{j = 1}^{{r_{\left[ i \right] }}} {{Y_j}} + \left( {r - {r_{\left[ i \right] }}} \right) {Y^*}} \right) } , \end{aligned}$$

and

17 $$\begin{aligned} V\left( \hat{\bar{Z}}_{R} \right) = {\left\{ \begin{array}{ll} \frac{1}{{nm}}\sum \limits _{i = 1}^m {\left( {{P_{\left[ i \right] }}\sigma _{z\left[ i \right] }^2 + rQ_{\left[ i \right] }^2\sigma _{z\left[ i \right] }^{*2}} \right) + \frac{2}{{{r^2}}}} \sum \limits _{i = 1}^{\frac{m}{2}} {\left( {{z\sigma _{\left[ {i,m + 1 - i} \right] }} + {r^2}Q_{\left( i \right) }^2\sigma _{z\left[ {i,m + 1 - i} \right] }^*} \right) } & \text {if}\, m\, \text {is even} \\ \frac{1}{{nm}}\sum \limits _{i = 1}^m {\left( {{P_{\left[ i \right] }}\sigma _{z\left[ i \right] }^2 + rQ_{\left[ i \right] }^2\sigma _{z\left[ i \right] }^{*2}} \right) + \frac{2}{{{r^2}}}} \sum \limits _{i = 1}^{\frac{{m - 1}}{2}} {\left( {{\sigma _{z\left[ {i,m + 1 - i} \right] }} + {r^2}Q_{\left( i \right) }^2\sigma _{z\left[ {i,m + 1 - i} \right] }^*} \right) } & \text {if}\, m\, \text {is odd} \\ \end{array}\right. } \end{aligned}$$

### Efficiency comparison

In this section, we provide mathematical conditions that rely on some measurable quantities for the data under consideration. If these conditions are satisfied, the proposed estimator will estimate the finite population mean more efficiently than

1. The naive approach, if
  18 $$\begin{aligned} {\left\{ \begin{array}{ll} \frac{{\frac{1}{m}\sum \limits _{i = 1}^m {\left( {{A_{\left( i \right) }} + r{B_{\left( i \right) }}} \right) + \frac{{2n}}{{{r^2}}}\sum \limits _{i = 1}^{\frac{m}{2}} {\left( {{C_{\left( i \right) }} + {r^2}{D_{\left( i \right) }}} \right) } } }}{{n{\lambda _n}\sigma _z^2 + {W_2}\left( {K - 1} \right) \sigma _{z2}^2}}< 1 & \text {if}\, m\, \text {is even} \\ \frac{{\frac{1}{m}\sum \limits _{i = 1}^m {\left( {{A_{\left( i \right) }} + r{B_{\left( i \right) }}} \right) + \frac{{2n}}{{{r^2}}}\sum \limits _{i = 1}^{\frac{{m - 1}}{2}} {\left( {{C_{\left( i \right) }} + {r^2}{D_{\left( i \right) }}} \right) } } }}{{n{\lambda _n}\sigma _z^2 + {W_2}\left( {K - 1} \right) \sigma _{z2}^2}} < 1 & \text {if}\, m\, \text {is odd} \\ \end{array}\right. } \end{aligned}$$
2. Bouza (2013) Approach, if
  19 $$\begin{aligned} {\left\{ \begin{array}{ll} \frac{{\sum \limits _{i = 1}^m {\left( {{A_{\left( i \right) }} + r{B_{\left( i \right) }}} \right) + \frac{{2nm}}{{{r^2}}}\sum \limits _{i = 1}^{\frac{m}{2}} {\left( {{C_{\left( i \right) }} + {r^2}{D_{\left( i \right) }}} \right) } } }}{{\sigma _z^2 + {W_2}\left( {K - 1} \right) \sigma _{z2}^2 - {W_2}K\sum \limits _{i = 1}^m {\Delta _{2\left( i \right) }^2} }}< 1 & \text {if}\, m\, \text {is even} \\ \frac{{\sum \limits _{i = 1}^m {\left( {{A_{\left( i \right) }} + r{B_{\left( i \right) }}} \right) + \frac{{2nm}}{{{r^2}}}\sum \limits _{i = 1}^{\frac{{m - 1}}{2}} {\left( {{C_{\left( i \right) }} + {r^2}{D_{\left( i \right) }}} \right) } } }}{{\sigma _z^2 + {W_2}\left( {K - 1} \right) \sigma _{z2}^2 - {W_2}K\sum \limits _{i = 1}^m {\Delta _{2\left( i \right) }^2} }} < 1 & \text {if}\, m\, \text {is odd} \\ \end{array}\right. } \end{aligned}$$
3. Fatima (2022) Approach, if
  20 $$\begin{aligned} {\left\{ \begin{array}{ll} \frac{{\sum \limits _{i = 1}^m {\left( {{A_{\left( i \right) }} + r{B_{\left( i \right) }}} \right) + \frac{{2nm}}{{{r^2}}}\sum \limits _{i = 1}^{\frac{m}{2}} {\left( {{C_{\left( i \right) }} + {r^2}{D_{\left( i \right) }}} \right) } } }}{{m\sigma _z^2 + \sigma _z^2\sum \limits _{i = 1}^m {{\omega _{2\left( i \right) }}\left( {{K_{\left( i \right) }} - 1} \right) } - \sum \limits _{i = 1}^m {{\omega _{2\left( i \right) }}\left( {{K_{\left( i \right) }} - 1} \right) \Delta _{2\left( i \right) }^2} }}< 1 & \text {if}\, m\, \text {is even} \\ \frac{{\sum \limits _{i = 1}^m {\left( {{A_{\left( i \right) }} + r{B_{\left( i \right) }}} \right) + \frac{{2nm}}{{{r^2}}}\sum \limits _{i = 1}^{\frac{{m - 1}}{2}} {\left( {{C_{\left( i \right) }} + {r^2}{D_{\left( i \right) }}} \right) } } }}{{m\sigma _z^2 + \sigma _z^2\sum \limits _{i = 1}^m {{\omega _{2\left( i \right) }}\left( {{K_{\left( i \right) }} - 1} \right) } - \sum \limits _{i = 1}^m {{\omega _{2\left( i \right) }}\left( {{K_{\left( i \right) }} - 1} \right) \Delta _{2\left( i \right) }^2} }} < 1 & \text {if}\, m\, \text {is odd} \\ \end{array}\right. } \end{aligned}$$

Where

$${A_{\left( i \right) }} = {P_{\left( i \right) }}\sigma _{\left( i \right) }^2$$, $${B_{\left( i \right) }} = Q_{\left( i \right) }^2\sigma _{\left( i \right) }^{*2}$$, $${C_{\left( i \right) }} = {\sigma _{\left( {i,m + 1 - i} \right) }}$$, and $${D_{\left( i \right) }} = \sigma _{\left( {i,m + 1 - i} \right) }^*$$

## Simulation study

A simulation study is conducted to evaluate the efficiency of the proposed method of subsampling for estimating the population mean compared to other existing methods. We first generate a bi-variate population (*Z*, *X*) with a predetermined correlation coefficient $\rho _{zx}$. This involves generating *X* values from a predefined distribution (normal and exponential) using R software, then producing *Z* values using the relationship $Z = {\rho _{zx}}X + \varepsilon \sqrt{1 - \rho _{zx}^2}$, where $\varepsilon$ is a standardized normal variable. Following the procedure of existing and proposed methods discussed above, we draw a sample of size $n=rm$ assuming some fixed response rate (RR). In accordance with the relevant procedures, a subsample of size $m(<n_2)$ is also selected, and the sample mean for the overall sample is computed. We repeat this procedure 2500 times to calculate the mean square error.

21 $$\begin{aligned} MSE({\hat{\bar{Z}}_\bullet }) = \frac{1}{{2500}}\sum \limits _{i = 1}^{2500} {{{\left( {{\hat{\bar{Z}}_\bullet } - {\mu _z}} \right) }^2}}, \end{aligned}$$

where $\hat{\bar{Z}}_\bullet$ is the estimated sample mean based on any estimator discussed above. This whole procedure is further repeated 2500 times to construct a plot displaying the $\varepsilon$ magnitude of MSE for an estimator against the probability of its occurrence. The x-axis shows the magnitude of MSE in terms of $\varepsilon$, while the probability of its occurrences is displayed on the y-axis. This plot helps to understand and compare the precision of estimates and the uncertainties associated with them; thus, we refer to this probability of achieving the $\epsilon$-precision, represented by

22 $$\begin{aligned} P\left( \varepsilon \right) = \Pr \left( {MSE\left( \hat{\bar{Z}}_\bullet \right) \leqslant \varepsilon } \right) . \end{aligned}$$

The term $P(\varepsilon )$ is the probability of achieving MSE less than or equal to the $\varepsilon$ value. Results are obtained for perfect ranking and imperfect ranking considering the following two controlled sampling setups:

1. **Setup-I:**$$n=30, r=10, m=3, RR=60\%$$.
2. **Setup-II:**$$n=50, r=10, m=5, RR=70\%$$.

The steps involved in the simulation study are summarized in the following algorithm:

**Algorithm 1:**
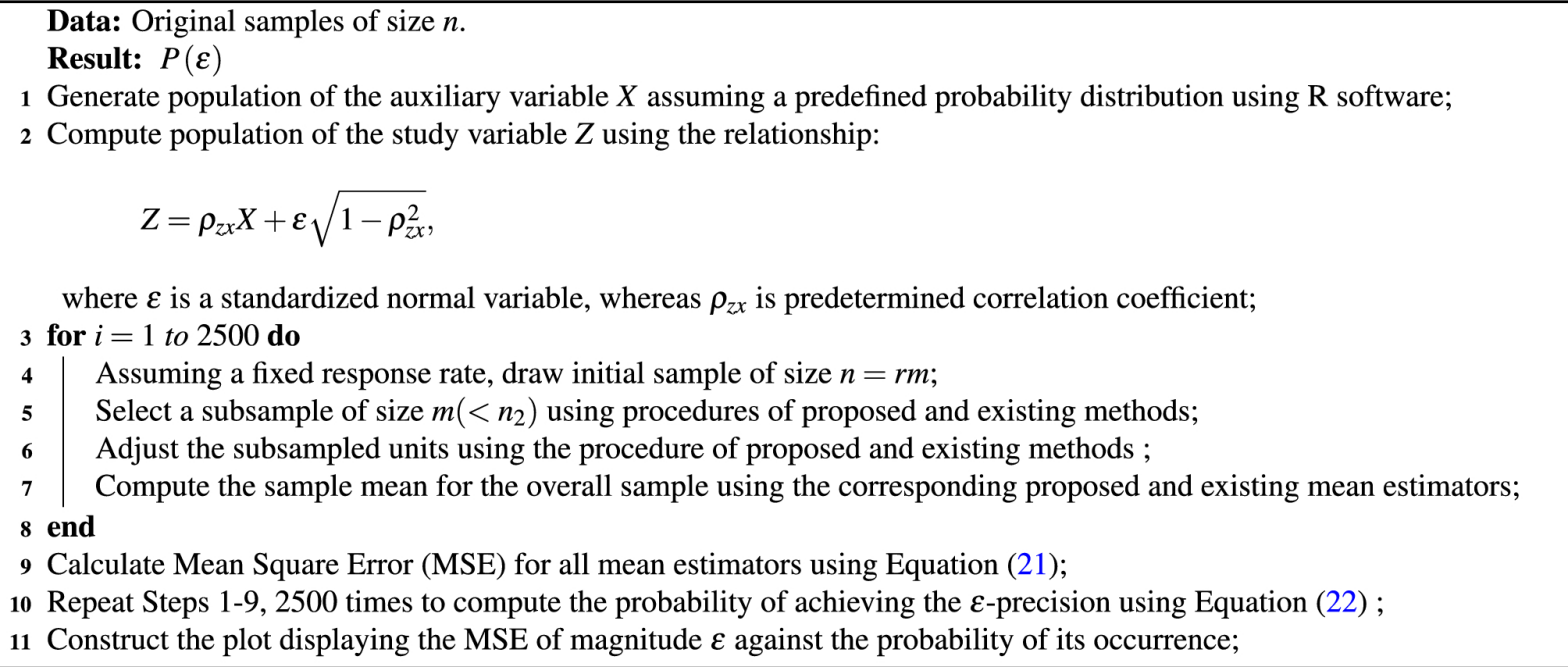
Determining probability of $\varepsilon$-precision.

**Fig. 2:**
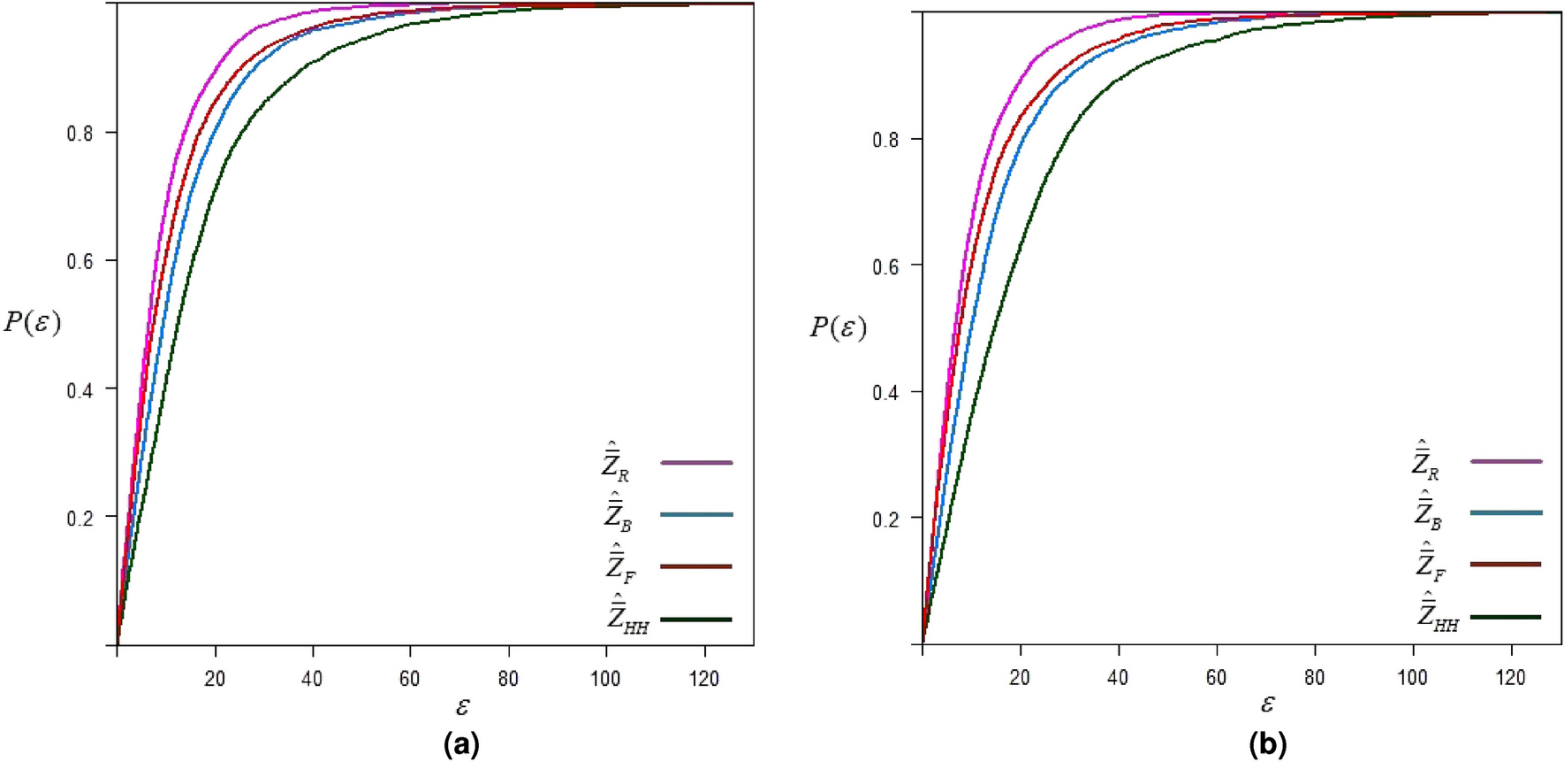
$\epsilon$-precision of different estimators for data setup-I under normal distribution.

**Fig. 3:**
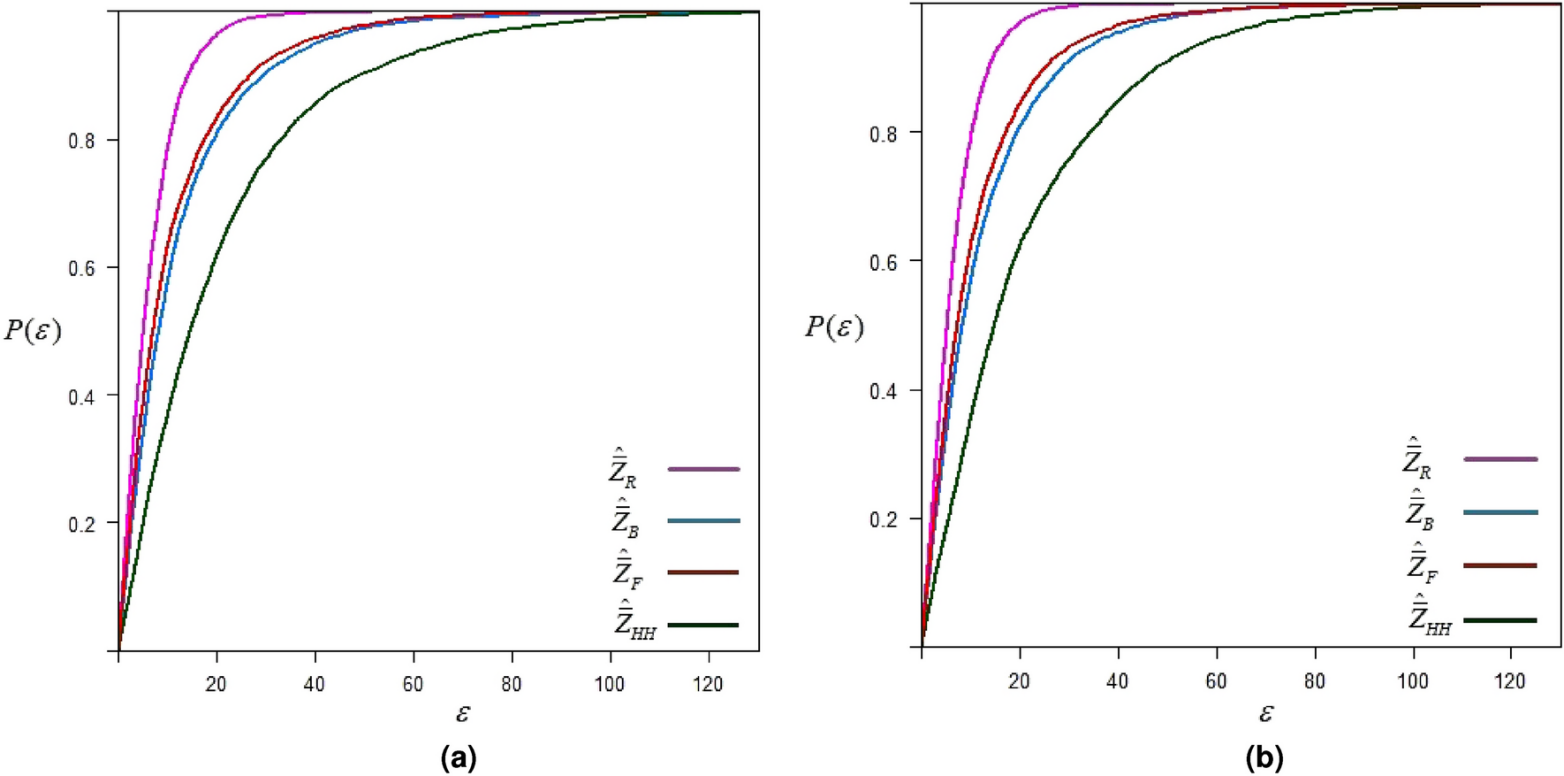
$\epsilon$-precision of different estimators for data setup-II under normal distribution.

**Fig. 4:**
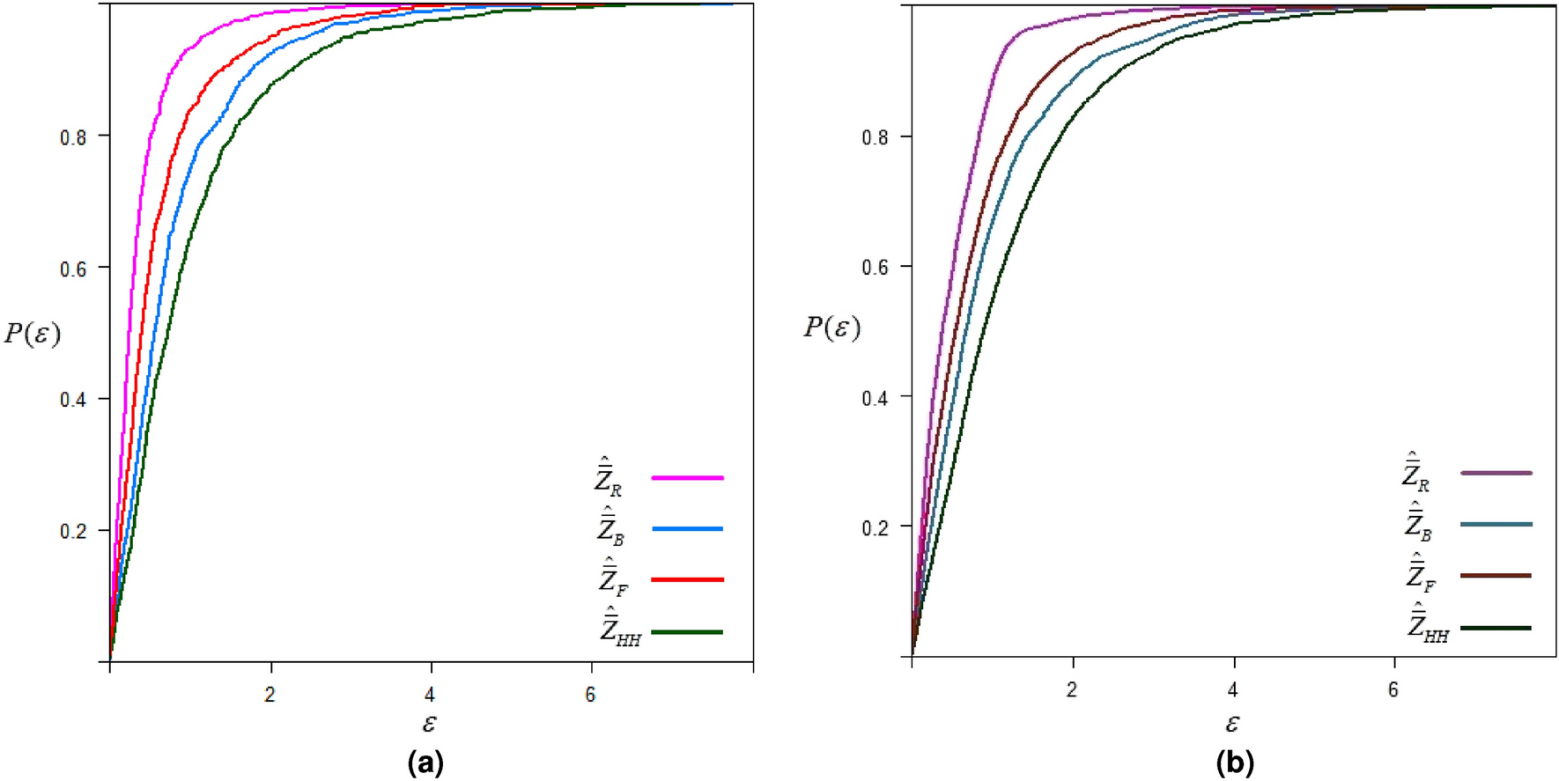
$\epsilon$-precision of different estimators for data setup-I under exponential distribution.

**Fig. 5:**
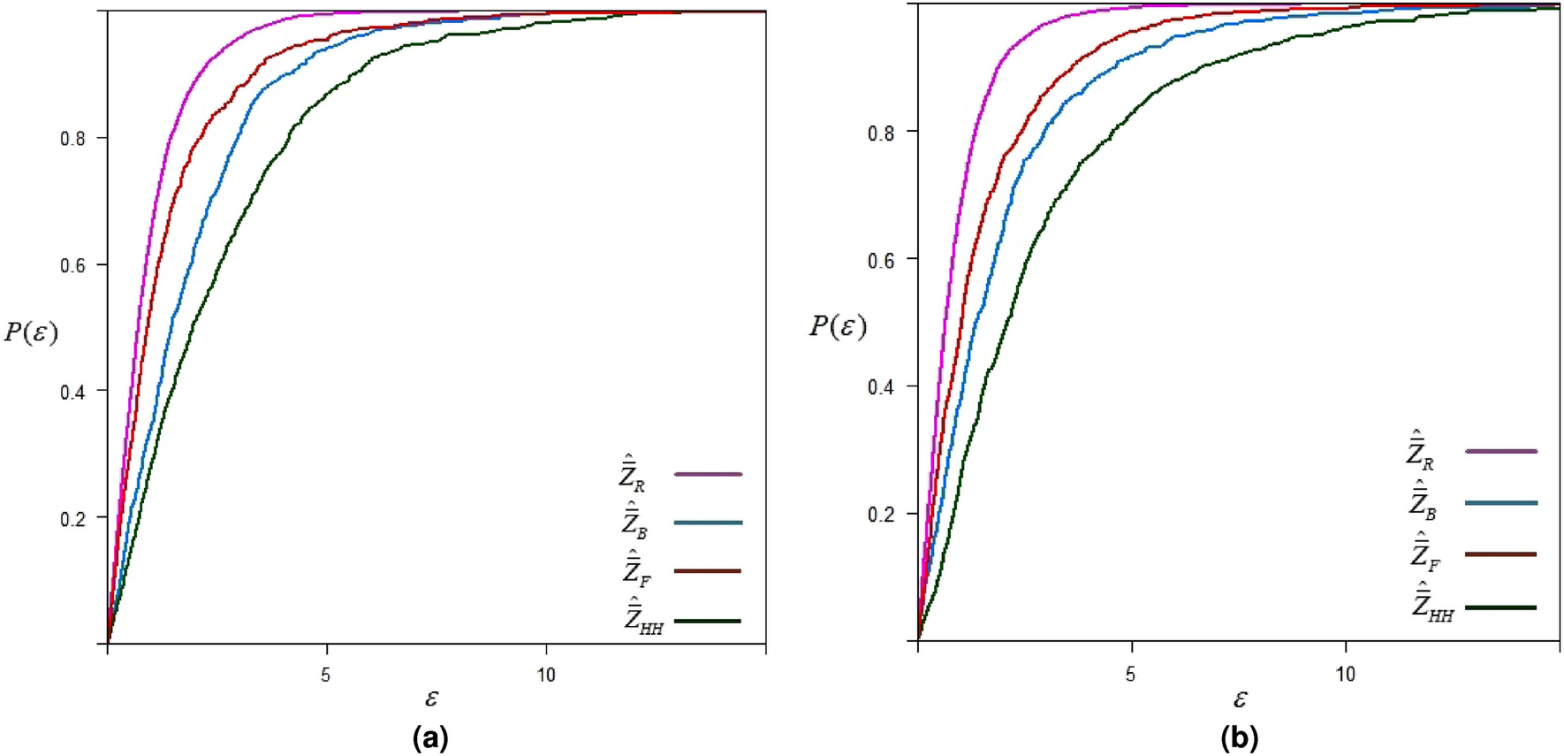
$\epsilon$-precision of different estimators for data setup-II under exponential distribution.

Simulation results are given below in Figs. 2, 3, 4 and 5.

## Application to real data

We consider data on abalone that was originally collected by^17^. Our goal is to estimate the population mean for the shucked weight of abalone, which is the total weight of meat after the shell is removed. The evasive nature of abalone can make sampling challenging and might result in non-response when data collection attempts are made. We consider the diameter of the abalone for ranking. The study variable *Y* and the concomitant variable *X* are determined as

*Y*= Shucked weight of abalone (grams).

*X*= Diameter of abalone (mm).

**Fig. 6:**
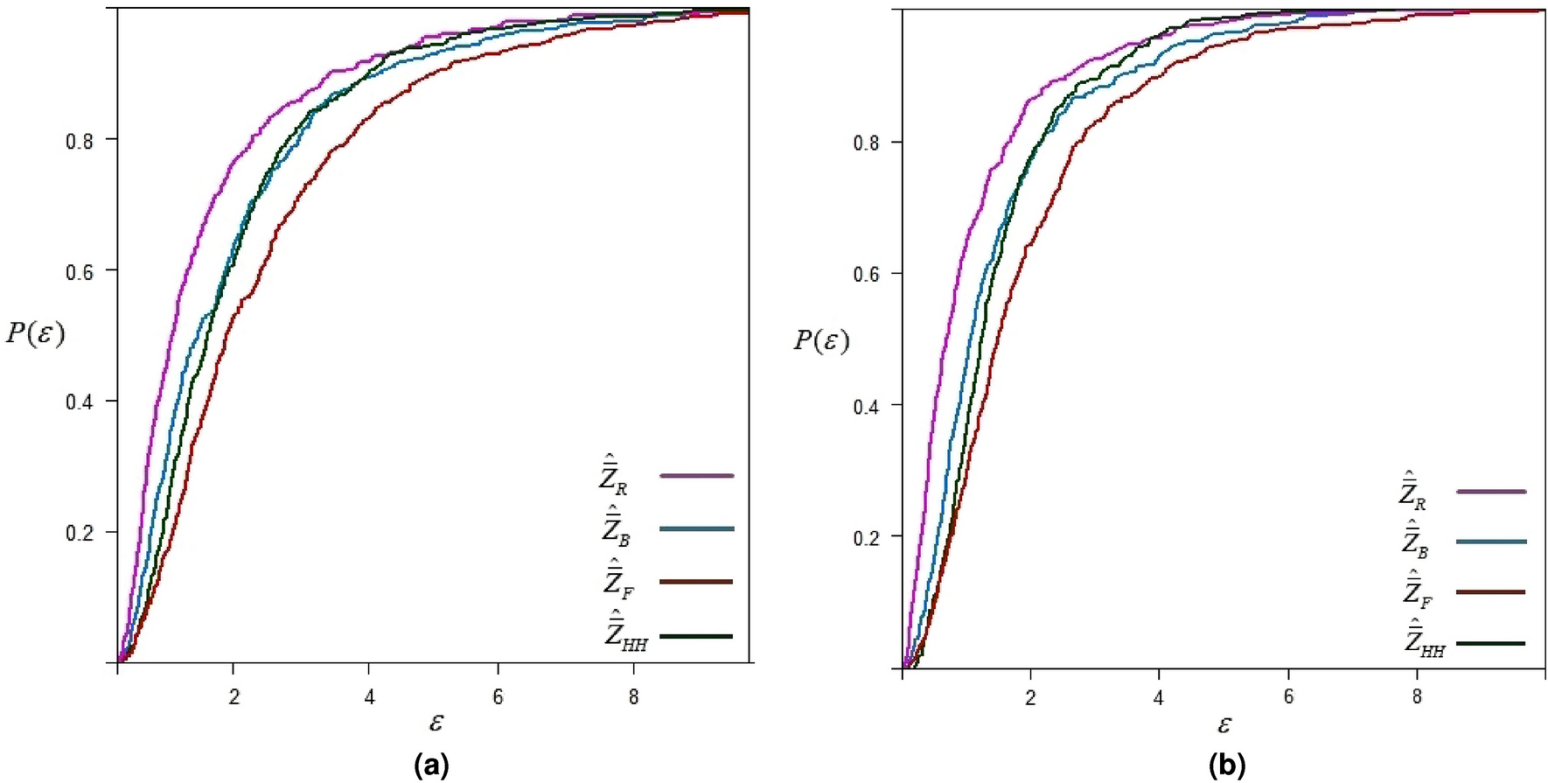
$\epsilon$-precision of different estimators for estimating shucked weight of abalone.

The simulation procedure is the same as discussed in “Simulation study” section. The simulation results for sampling setup-I and sampling setup-II are given below in Fig. 6.

## Discussions

Simulation results given in Figs. 2-5 show that the proposed mean estimator $\hat{\bar{Z}}_R$ yields more precise estimates of the population mean when compared to other existing estimators, i.e., the proposed estimator has the highest probability of achieving the desired MSE of magnitude $\varepsilon$. We observe comparatively higher precision in the estimates of the suggested estimator when the total sample size *n* is increased. It is also observed that the precision is higher in the case of an exponential distribution when compared with a normal distribution. Additionally, in the case of exponential distribution, the precision of the suggested estimator is higher under perfect ranking as compared to the case of imperfect ranking.

The results of the simulation study based on abalone data, as given in Fig. 6, show similar trends in the precision of estimates as obtained for hypothetical normal and exponential distributions. The proposed estimator has a greater chance of estimating the population mean within the range of $\varepsilon$-precision.

## Conclusions

In this paper, we proposed a novel estimator for the finite population mean in the presence of non-response by utilizing the Paired Ranked Set Sampling (PRSS) technique. The sampling procedure that provides the basis for developing the proposed estimator is explained, and the procedure for adapting subsampling units into the initial sample is described. We derive the expression for the variance of the proposed estimator and establish conditions under which it estimates the population mean more precisely than other existing estimators. A comprehensive simulation study is conducted to compare the precision of the suggested estimator with other existing estimators, considering normal distribution, exponential distribution, and real-life data on abalone. The simulation results show that the proposed estimator is more likely to produce estimates within a given $\varepsilon$ range. Based on this study, we recommend using the proposed method of subsampling and the associated estimator for the estimation of the population mean in scenarios involving non-response.

This study could be extended to develop more praised estimators such as ratio, product, and regression estimators that utilize the known information on the auxiliary variable for estimating the population mean of the study variable. Additionally, the strategy of adopting subsampled units into the initial sample can be extended to develop estimators of population variance and other parameters.

## Data Availability

The data used in this study is publicly available from the UCI Machine Learning Repository at the URL: https://archive.ics.uci.edu/dataset/1/abalone.
